# Evaluation of muscle quality and quantity for the assessment of sarcopenia using mid-thigh computed tomography: a cohort study

**DOI:** 10.1186/s12877-021-02187-w

**Published:** 2021-04-13

**Authors:** Hiroki Oba, Yasumoto Matsui, Hidenori Arai, Tsuyoshi Watanabe, Hiroki Iida, Takafumi Mizuno, Satoshi Yamashita, Shinya Ishizuka, Yasuo Suzuki, Hideki Hiraiwa, Shiro Imagama

**Affiliations:** 1 Department of Orthopedics, Nagoya University Graduate School of Medicine, 65 Tsurumaicho, Showaku, Nagoya, Aichi 466-8560 Japan; 2 Center for Frailty and Locomotive Syndrome, National Center for Geriatrics and Gerontology, 7-430 Moriokacho, Obu, Aichi 474-8511 Japan; 3 Department of Orthopedics, National Center for Geriatrics and Gerontology, 7-430 Moriokacho, Obu, Aichi 474-8511 Japan; 4 Department of Human Care Engineering, Faculty of Health Sciences, Nihon Fukushi University, Okuda, Mihamacho, Chita, Aichi 470-3295 Japan

**Keywords:** Sarcopenia, Mid-thigh computed tomography, Muscle quality, Computed tomography attenuation value, Motor function

## Abstract

**Background:**

For the diagnosis of Sarcopenia, European Working Group on Sarcopenia in Older People (EWGSOP) revised the algorisms in 2019, where they added computed tomography (CT) as an assessment tool not only for quantity but also for quality in research purpose. However, the evidence for clinical appreciation of CT has been lacking. Therefore, we investigated the correlation between CT and various motor function tests to assess the utility of CT as a potential diagnostic tool for sarcopenia.

**Methods:**

In total, 214 patients who were examined at our center during the study period (2016–2017) were included in the study. Single-slice CT scan of the mid-thigh region was performed, from which cross-sectional area (CSA) and CT attenuation value (CTV) of quadriceps femoris were evaluated for each subject. Other assessments included skeletal muscle mass index by DXA and BIA, muscle strength and physical performance. Furthermore, subjects were classified into four groups as per the Asia Working Group of Sarcopenia (AWGS) 2019 criteria as those with: normal, poor muscle function/strength (poor function), sarcopenia and severe sarcopenia.

**Results:**

Knee muscle strength correlated with CSA (r = 0.60) and the correlation was significantly greater than that with DXA and BIA. For physical performance, standing-up test correlated with CSA (r = − 0.20) and CTV (r = − 0.40) and walking speed with CTV (r = 0.43), which was significantly greater than that with DXA and BIA. The CSA was significantly lower in women with sarcopenia group and in both men and women with severe sarcopenia (all *p* < 0.01). Furthermore, CTV was significantly lower in women with poor-function and in both men and women with severe sarcopenia group (all p < 0.01).

**Conclusions:**

CSA mostly correlated with muscle strength, whereas CTV mostly correlated with physical performance. CT with measurements of CSA and CTV enables the evaluation of muscle mass and quality simultaneously. CT is believed to be useful in inferring evaluation of motor function and assessment of sarcopenia.

## Background

As the adult population ages, the extension of healthy life expectancy has become a major issue. Sarcopenia was initially defined as a reduction in the skeletal muscle mass with aging. The term “sarcopenia” was first introduced by Rosemberg et al. [1] in 1989, from the Greek terms “sarx” (muscle) and “penia” (loss). In 1998, Baumgartner et al. [2] defined sarcopenia as a mean skeletal muscle mass index (SMI) of −2SD or less determined using dual energy X-ray absorption (DXA) in young individuals, and since then, sarcopenia has been determined using SMI. However, it has been reported that compared with muscle mass, muscle strength is more associated with falling and limited mobility [3, 4], and therefore, in determining sarcopenia, it is thought that muscle strength and physical performance should be included. The 2010 consensus report from the European Working Group on Sarcopenia in older people (EWGSOP) [5] proposed a diagnostic algorithm for determining sarcopenia according to SMI, grip strength, and walking speed. In 2011, a condition “sarcopenia with limited mobility” defined as a person with muscle loss whose walking ability is reduced was described as a condition that requires treatment [6].

In 2014, the Asia Working Group of Sarcopenia (AWGS) presented diagnostic criteria for sarcopenia based on data obtained from various Asian countries [7]. Furthermore, In the EWGSOP2 revised in 2018, attention was focused on loss of muscle strength as the primary parameter of sarcopenia, and for a definite diagnosis of sarcopenia, it was reported that the assessment of muscle quantity and quality is important [8]. AWGS 2019 updated the consensus of sarcopenia diagnosis in Asia: the previous definition of sarcopenia was retained; however, the diagnostic algorithm, protocols, and some criteria have been revised. Specifically, AWGS 2019 introduces “possible sarcopenia,” defined by low muscle strength with or without reduced physical performance [9].

Muscle mass is conventionally measured using DXA and bioelectrical impedance analysis (BIA). DXA can measure appendicular skeletal muscle mass; however, there are problems with this method, i.e., the values differ depending on the machine used [10], and in Asian women, this measurement does not change with age [11]. BIA is affected by the amount of body fluids; therefore, precise evaluations cannot be achieved [12]. Therefore, the EWGSOP2 described computed tomography (CT) and magnetic resonance imaging (MRI) as methods to verify the muscle quantity and quality; however, there are only few reports of their use in clinical practice [13].

Motor function is evaluated using measurements of walking speed and grip strength; however, the reproducibility is poor depending on the physical and mental condition of the participant and the measurement methods and tool used [14]. CT and MRI are considered the gold standards of minimally invasive tests to measure muscle mass [15]; however, their use in the evaluation of muscle quality remains controversial. Goodpaster et al. [16] noted a good correlation between CT attenuation value (CTV) and intramuscular fat infiltration, whereas Ikemoto-Uezumi et al. [17] reported reduced muscle fibers and fatty tissue infiltration in the muscular tissue of the vastus medialis in patients with osteoarthritis. Therefore, it is inferred that CT can evaluate muscle quantity and that CTV can evaluate fat infiltration which might be associated with decreased motor function.

However, there are few reports of the use of CTV in CT scans of the mid-thigh and their use in evaluating motor function. The purpose of this study was to evaluate to what extent CT, DXA and BIA derived muscle parameters correlate with muscle strength and function in order to investigate the utility of CT as a potential assessment method for muscle quality as well as for the assessment of sarcopenia.

## Methods

Of 300 patients who were examined at the outpatient services of the National Center for Geriatrics and Gerontology, Center for Frailty and Locomotive Syndrome between 2016 and 2017, 214 (78 men and 136 women; average age, 78.3 and 78.4 years respectively) were included in the study after excluding those with gait impairment caused by severe osteoarthritis of the knee and hip, progressive motor disease such as Parkinson disease, dementia and those with missing data. To assess the risk of falls, we investigated fall scores, as proposed by Toba et al. [18] To assess motor function, we measured walking speed (Walkway MW-1000, ANIMA, Tokyo Japan); grip strength using a new grip dynamometer developed at our institution with ZP-500 N (Imada Co., Ltd., Toyohashi, Aichi, Japan) [19]; knee extension strength (ZP-500 N, Imada Co., Ltd., Toyohashi, Aichi, Japan) that is the measure of the isometric muscle extension strength in the sitting position with knee at 90° of flexion, developed at our institution [20]; single-leg standing; timed up and go (TUG); short physical performance battery (SPPB) using stand-up test and two-step test. For single leg standing, the participants were instructed to stand on a single leg with their eyes open as long as possible and the time was recorded. For TUG, we measured the time required to stand up from the chair and go back 3 m and to sit on the chair again. The stand-up and two-step tests were performed to assess the degree of locomotive syndrome. For the stand-up test, we measured whether the participants could stand up on both the legs from the sitting position on a platform of 10, 20, 30, and 40 cm [21]. For the two-step test, the participants stood at the starting line and took two strides as long as possible; then, the two legs were aligned and the distance of the two steps was measured. For SPPB, the test evaluated the balance, gait, strength, and endurance by examining an individual’s ability to stand on their feet together in side-by-side semi-tandem and tandem positions, time to walk 8 ft., and time to rise from a chair and return to the seated position five times [22]. Furthermore, the muscle mass of the four limbs from the whole-body scan was measured by DXA (Lunar iDXA, GE Healthcare, Chicago, Illinois, USA) and BIA (Inbody720, BIOSPACE, Iowa, USA). Appendicular skeletal muscle mass (ASM) (kg) by DXA and BIA / height^2^(m^2^) was calculated as a skeletal muscle mass index (SMI) [2].

**Fig. 1:**
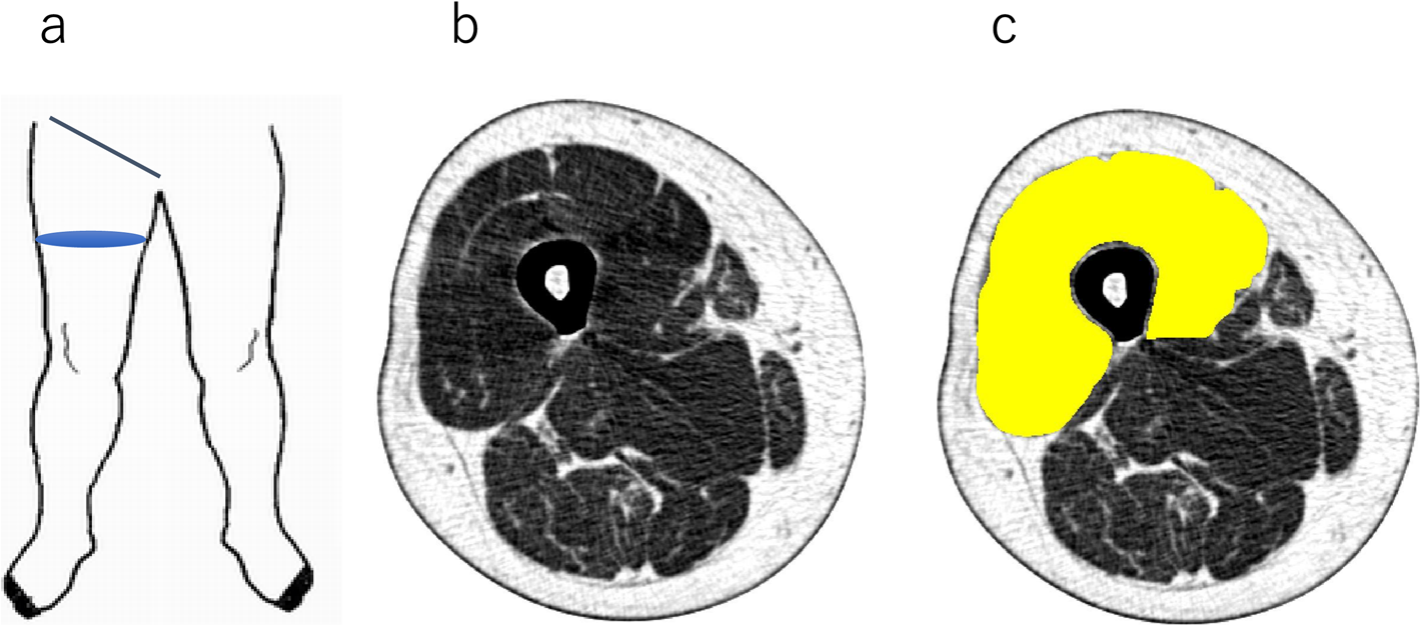
Procedure for CT measurement. **a.** We defined the mid-thigh as the midpoint of the inguinal ligament and the superior pole of the patella manually. **b**. Obtained CT image of mid-thigh. **c**. Trace quadriceps femoris using SliceOmatic manually. CT: computed tomography

For CT (SOMATOM Sensation 64; Siemens, Munich, Germany), the subject was placed in the supine position, and a single slice of the right mid-thigh was taken. We defined the mid-thigh as the midpoint of the inguinal ligament and the superior pole of the patella manually. The scanning conditions were as follows: 120 kV, 120 mA, rotation time of 1 s, and field of view of 233 mm. From this single slice, the cross-sectional surface area (CSA) and CTV of the quadriceps femoris muscle were measured using SliceOmatic ver.5.0 (Tomovision, Canada) by a single examiner (Fig. 1). CTV was defined as − 1000 Hounsfield Unit (HU) for air and 0 HU for water and indicated the degree of X-ray absorption of a substance. The average CTV is 40–100 HU for a muscle and approximately − 50 to − 100 HU for fat. For CSA and CTV, the imaging data of 21 subjects were measured by three examiners, and the associated interclass correlation was determined. The intraclass correlation of CSA and CTV of the examiners was > 0.998, and the interclass correlation among the three examiners was 0.999.

The subjects were classified in accordance with the AWGS 2019 diagnostic criteria [7] into four groups. Among the subjects without reduced walking speed and grip strength, the group with normal muscle mass was defined as the “normal” group and the group with reduced walking speed and/or grip strength and normal muscle mass was defined as the “poor muscle function/strength (poor function)” group. The group with low ASM and low muscle strength or low physical performance was termed the “sarcopenia” group, and the group with low ASM, low muscle strength, and low physical performance was called the “severe-sarcopenia” group.

According to the AWGS 2019 criteria, the cutoff values [7] were as follows: walking speed,1.0 m/s; grip strength,28 kg for men and 18 kg for women; SMI with DXA 7.0 kg/m^2^ for men and 5.4 kg/m^2^ for women; and SMI with BIA 7.0 kg/m^2^ for men and 5.7 kg/m^2^ for women. For the four groups, we evaluated the association between the muscle function and CT, DXA and BIA.

Statistical analysis were performed via SPSS (IBM SPSS Statistics ver.24, Tokyo, Japan) using ANOVA for demographic data for men and women, CSA and CTV of the quadriceps femoris muscle between each four groups. Tukey’s test was applied for post-hoc multiple testing. To determine the association between muscle strength and function parameters on one hand, and CSA, CTV, DXA and BIA on the other hand, partial correlations were calculated to correct for the effect of gender. Pearson’s correlation coefficient was applied for muscle strength and Spearman’s correlation coefficient was applied for physical performance test. The difference of correlation coefficient among the subjects was calculated using Fisher’s Z transformation [23], and then Bonferroni correction was performed. *P* values of < 0.05 were considered statistically significant. Power analysis was performed via G*Power (Heinrich Heine university, version 3.1.9.7, Dusseldorf, Germany). The parameters were as follows: α = 0.05, 1-β = 0.8, assumed correlation coefficients between CSA and grip strength was 0.2, CTV and grip strength was 0.4 and CTV and CSA was 0.3 [24]. Because the calculated appropriate sample size was 236, the sample size was almost sufficient for the study.

## Results

Overall, 99 subjects were included in the normal group, 55 in the poor-function group, 26 in the sarcopenia group, and 34 in the severe sarcopenia group. Compared with the normal group, age and the fall risk index were significantly higher in the poor-function, severe sarcopenia groups in women (Table 1).

**Table 1:** Demographic data by group

Group				Age (year)		Height (cm)		Weight (kg)		BMI (kg/cm^2^)		Fall risk score	
	n			average	SD	average	SD	average	SD	average	SD	average	SD
Normal	99	M	36	77.0	5.0	163.6	4.7	60.8	7.0	22.7	2.5	7.3	3.1
		F	63	75.6	5.1	150.7	5.4	52.3	9.7	23.0	3.9	7.9	3.2
Poor-function	55	M	13	77.7	5.1	159.3	8.5	67.9	12.1	26.6	3.1	9.2	3.2
		F	42	78.9*	5.3	146.7	7.2	53.9	10.1	25.0*	3.8	11.0*	3.0
Sarcopenia	26	M	14	79.9	5.8	159.9	4.9	55.6	7.6	21.7	2.2	8.8	2.9
		F	12	81.8*	3.5	148.7	4.9	41.5*	5.6	18.8*	2.5	10.4*	2.2
Severe Sarcopenia	34	M	15	81.6	7.9	161.6	7.7	54.1	9.7	20.7	3.5	10.1*	3.4
		F	19	83.6*	4.7	143.6	5.7	39.6*	5.6	19.1*	1.6	10.7*	2.8
Total	214	M	78	78.5	6.0	161.8	6.3	59.7	9.6	22.8	3.3	8.4	3.3
		F	136	78.3	5.7	148.3	6.5	50.1	10.5	22.7	4.1	9.5*	3.3

*P < 0.05 compared with normal group, *M* men, *W* women, *BMI* body mass index, *SD* standard deviation

Age was significantly higher in the poor-function, sarcopenia and severe-sarcopenia groups than in the normal group

Knee extension strength and grip strength were correlated with CSA, BIA, DXA and CTV. CSA had significantly stronger correlation with knee extension strength than BIA and DXA, whereas CTV did not have significantly stronger correlation with others (Table 2).

**Table 2:** Correlation between each method and motor function (muscle strength and physical performance) test

	CSA			BIA			DXA			CTV		
	r	95%CI		r	95%CI		r	95%CI		r	95%CI	
		Lower	Upper		Lower	Upper		Lower	Upper		Lower	Upper
Muscle strength												
Knee extension strength	0.60	0.56	0.83	0.391*	0.28	0.55	0.39*	0.27	0.54	0.26	0.13	0.40
Grip strength	0.44	0.34	0.61	0.29	0.16	0.43	0.39	0.28	0.55	0.26	0.13	0.40
Physical performance												
Single-leg standing	0.24	0.11	0.36	0.18	0.05	0.31	0.20	0.07	0.33	0.24	0.11	0.37
Walking speed	0.34	0.21	0.45	0.22†	0.09	0.35	0.29	0.17	0.41	0.43	0.31	0.53
Standing up test	−0.20	−0.33	−0.07	0.01*†	−0.12	0.15	0.05*†	−0.08	0.19	−0.40	− 0.51	− 0.28
TUG	− 0.38	− 049	− 0.26	−0.27	− 0.40	−0.14	− 0.30	−0.42	− 0.18	−0.34	− 0.45	−0.21
SPPB (total)	0.36	0.24	0.48	0.23	0.10	0.36	0.26	0.13	0.38	0.35	0.22	0.46
Two-step test	0.33	0.21	0.45	0.24	0.11	0.37	0.31	0.19	0.43	0.40	0.28	0.51

*CSA* cross-sectional area of quadriceps femoris measured by computed tomography (CT) scan, *CTV* CT attenuation value of quadriceps femoris measured by CT scan, *r* Pearson’s partial correlation coefficient for muscle strengths and Spearman’s partial correlation coefficient for muscle performances, *BIA* Skeletal muscle Mass Index (SMI) determined by bioelectrical impedance analysis, *DXA* SMI determined by dual energy X-ray absorption

*P < 0.05 compared with CSA, †*P* < 0.05 compared with CTV. * or† means that CSA or CTV has significantly stronger correlation than the value. CSA showed the most association with muscle strength, whereas CTV showed the most association with physical performance

In contrast, in terms of the physical performance measurements, the correlation coefficients for CSA and CTV showed that these were correlated with single leg standing, walking speed, standing up test, TUG, SPPB, and two-step test. CTV showed a significantly stronger correlations with walking speed compared with BIA, and a significantly stronger correlation with the standing up test compared with both BIA and DXA (Table 2).

**Fig. 2:**
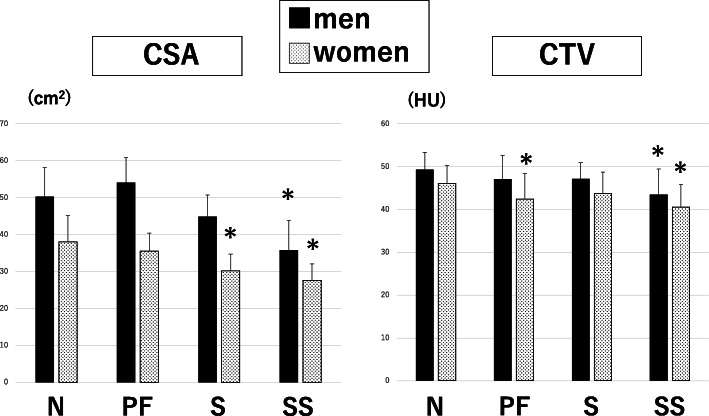
CSA and CTV of quadriceps femoris in each group. *P < 0.01 compared with normal group. CSA: cross-sectional area, CTV: computed tomography attenuation value, N: normal, PF: poor muscle function/strength, S: sarcopenia, SS: severe sarcopenia, HU: Hounsfield unit. CSA was significantly smaller in the S for women and SS for both men and women, whereas CTV was significantly lower in the PF in women and in the SS group for men and women

About CSA and CTV, three abnormal groups (poor-function, sarcopenia and severe sarcopenia groups) were compared with normal group. CSAs of the quadriceps femoris muscle were significantly smaller in the severe sarcopenia groups (*P* < 0.001) for men and significantly smaller in the sarcopenia (P < 0.001), and severe sarcopenia groups (P < 0.001) for women (Fig. 2). Whereas, CTVs were significantly lower in the severe sarcopenia group (*P* = 0.001) for men and significantly lower in the poor-function (*P* = 0.008) and severe sarcopenia groups (P < 0.001) for women (Fig. 2).

## Discussion

In this study, we determined the correlation between CT and various motor function, using different assessment methods for muscle quantity / quality, including CT, BIA, and DXA. The result indicated that CSA showed the most correlation with muscle strength, whereas CTV showed the most correlation with physical performance.

The concept of sarcopenia has considerably changed over time, and recently, the EWGSOP2 has stated that the importance of measuring muscle quality is expected to grow as a defining feature of sarcopenia; however, there is no universal consensus on the assessment method for routine clinical practice [8]. Patients with dynapenia and sarcopenia which are considered as conditions of low muscle quality are at a high risk of falling [3]. In accordance with the present study, patients with poor function and sarcopenia had a significantly higher risk of falling, which was believed to be associated with reduced motor function. There is a physical state in which muscle mass is maintained but with reduced functioning and muscle quality. McGregor et al. [25] referred that not only changes in muscle mass but also other factors underpinning muscle quality including composition, metabolism, aerobic capacity, insulin resistance, fat infiltration, fibrosis, and neural activation may play a role in the decline in muscle function and impaired mobility associated with aging.

To date, muscle quality is determined by evaluating intramuscular fat infiltration using highly sensitive measurement devices such as CT and MRI [26]. Moreover, in the recent years, muscle quality has been evaluated using ultrasound; however, there is no consensus for any of these assessments. There are few reports wherein CT of the mid-thigh has been used to evaluate the relationship of CSA and CTV using motor function. The present study was performed to evaluate whether CTV correlates with motor function, and whether it can be used to evaluate muscle quality.

In previous studies, parameters of simple muscle strength, such as grip [27] and quadriceps femoris muscle strength [28], have been correlated with CSA; in a similar manner, our study showed that the strongest correlation was that of CSA with grip and knee extension strength. Furthermore, in the evaluation of physical performance test that combined physical movement (single-leg standing, walking speed, stand-up test, TUG, SPPB, and two-step test), the highest correlation was observed with CTV, and walking speed and stand-up test showed a significantly stronger correlation with CTV than other inspection devices, suggesting that physical performance is associated with muscle quality as determined by CTV.

Lang et al. [29] reported that a low CTV of the thigh indicated a higher risk of fall and hip fracture; the results of the present study are consistent with these results. In this study, we classified patients into four groups as per muscle mass and motor function, based on the AWGS 2019 criteria. Between the women in the normal and sarcopenia groups, there was no decrease in the CTV; however, there was a significant decline in the CTV of those in the poor-function and severe sarcopenia groups. We consider that the decrease in CTV may be attributable to muscle atrophy and increased fat composition in the muscle. Ikemoto-Uezumi et al. [17] compared tissue in the vastus medialis muscle of patients with osteoarthritis and reported that intramuscular adipose tissue (IMAT) and an increased proportion of interstitial tissue were observed. Thus, a decline in CTV with an increased proportion of IMAT was associated with intramuscular fat infiltration [30]. Similarly, in sarcopenia, increased IMAT is observed [16], and these changes in muscle tissue could explain the decrease in CTV. Reportedly, increased IMAT measured by MRI is a prognostic factor of gait ability [31], and IMAT accumulates markedly after reduced activity in healthy young adults [32]. We consider that reduced activity or motor function may have caused an increase in IMAT and that CTV could evaluate this change in muscle composition.

As a minimally invasive tool, CT is considered as a gold standard to measure muscle quantity; however, it is not used in general practice owing to the high costs, poor portability, and requirement of an experienced operator [15]. In patients with cancer, reduced muscle mass is an independent predictor of immobility and mortality [33]. Kasai et al. [34] reported that among the muscles in the mid-thigh CT, CSA decreased with age mainly in quadriceps femoris. Schweitzer et al. [35] reported that on MRI, the best estimates for skeletal muscle were shown in the thigh. Lee SJ reported that CT of the femur was a useful method to evaluate muscle mass for the entire body [36]. Compared with other test equipment, DXA showed no change with age among Asian women [11]. Recently, we reported significant associations of thigh CT CSA with muscle strength [37]. For BIA, changes associated with the physical condition such as fluid balance and body temperature fluctuated during the day, resulting in reduced accuracy of BIA [38]. CT could be a better form of assessment because of its accuracy, reproducibility, and objectivity [39]. CT can simultaneously measure the muscle CSA and CTV, indicating muscle mass and composition; thus, CT is useful for assessing the severity of sarcopenia. Regarding the amount of radiation exposure, as only a single slice is scanned, we consider that there is no problem in terms of the radiation levels [10]. In addition, single slice leg CT scanning takes only about 5 min. We think that the present results represent the initial steps toward the accumulation of evidence for the evaluation of muscle quality and will form the basis for future studies to examine the pathophysiology of severe sarcopenia accompanied by not only muscle mass loss but also physical functional decline. It is also useful for detailed evaluation in patients who have difficulty to walk, or to confirm the muscle condition after screening with a simple physical test.

There were limitations to the present study. First, this was a cross-sectional study and changes caused by natural history or therapeutic intervention could not be evaluated. Further study is required to determine whether or not the changes occurred as a result of exercise therapy, nutritional counseling, and pharmacotherapy. Second, this study did not include large-scale data of CSA and CTV from each age group of the general population without sarcopenia, and therefore, the healthy control information was weak. Thus, analysis using a large-scale epidemiological study is required. In the future, we plan to perform an analysis on a larger sample. As noted in the EWGSOP2, our results showed that CT may be useful in objectively evaluating sarcopenia more accurately and understand the pathology in greater detail [8].

## Conclusions

This study was the first to report a relationship between mid-thigh CT and motor function in clinical setting. CT allows CSA and CTV to be measured simultaneously; CSA showed the most association with muscle strength, whereas CTV showed the most association with physical performance. These results could be useful for the assessing the presence and severity of sarcopenia.

## Data Availability

The datasets generated during and/or analyzed during the current study are available from the corresponding author on reasonable request.

## Abbreviations

AWGS: Asia Working Group of Sarcopenia
BIA: Bioelectrical impedance analysis
CSA: Cross-sectional area
CT: Computed tomography
CTV: CT Attenuation value
DXA: Dual energy X-ray absorption
EWGSOP: European Working Group on Sarcopenia in Older People
MRI: Magnetic resonance imaging
SMI: Skeletal muscle mass index
SPPB: Short physical performance battery
TUG: Timed Up and Go
HU: Hounsfield unit
